# Supplementary material for: Mapping the Literature on Nutritional Interventions in Cognitive Health: A Data-Driven Approach
Source: Nutrients. 2018 Dec 24;11(1):38. doi: 10.3390/nu11010038 (PMC6356193; doi:10.3390/nu11010038)
Supplement: Supplementary file 1 [file nutrients-11-00038-s001.zip › supplementary.docx]

**Supplementary Table 1.** Cluster 1: *n* = 3648 publications, topic of the association between diet and cognitive outcomes (focus on prediction of decline and disease)

| 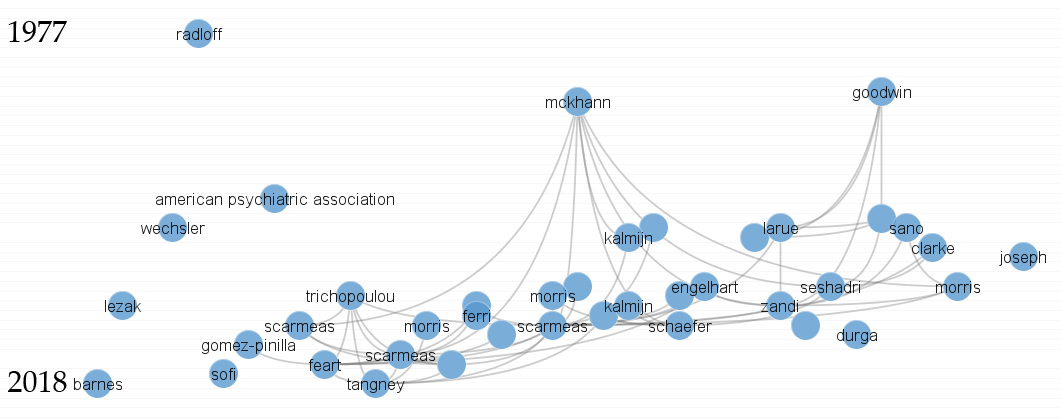 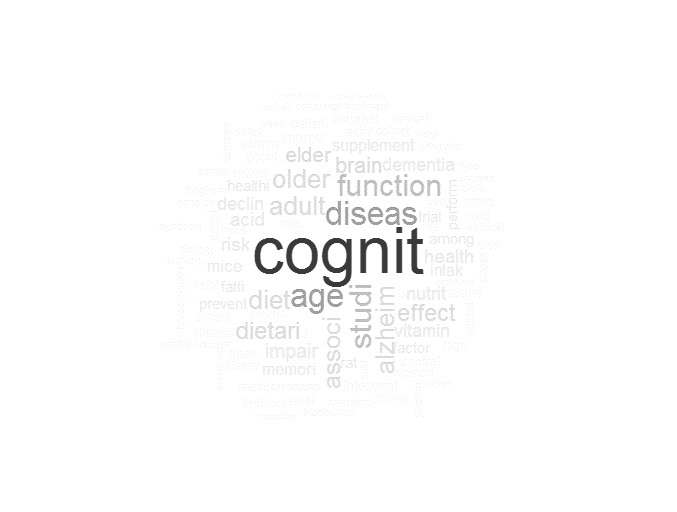 | | | | |
| --- | --- | --- | --- | --- |
| **Cluster information and indicative core publication (weighted for recency)** |  | **Word cloud** |  | **Most unique topic terms (β log ratio)** |
| **1a: *n*=3356, topic of cognition in ageing.** McKhann, G., Drachman, D., Folstein, M., Katzman, R., Price, D., & Stadlan, E. M. (1984). Clinical diagnosis of Alzheimer's disease Report of the NINCDS‐ADRDA Work Group* under the auspices of Department of Health and Human Services Task Force on Alzheimer's Disease. Neurology, 34(7), 939-939. doi: 10.1212/WNL.34.7.939 |  | 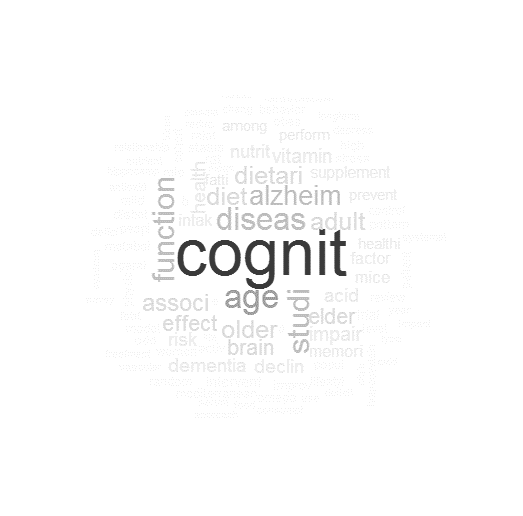 |  | prolifer* (8.84) disodium (8.41) nonalzheim* (8.38) rhodiola* (7.25) positron (7.02) |
| **1aa: n=3342, topic of dietary patterns in ageing.** Scarmeas, N., Stern, Y., Tang, M. X., Mayeux, R., & Luchsinger, J. A. (2006). Mediterranean diet and risk for Alzheimer's disease. Annals of Neurology: Official Journal of the American Neurological Association and the Child Neurology Society, 59(6), 912-921. doi: 10.1002/ana.20854 |  | 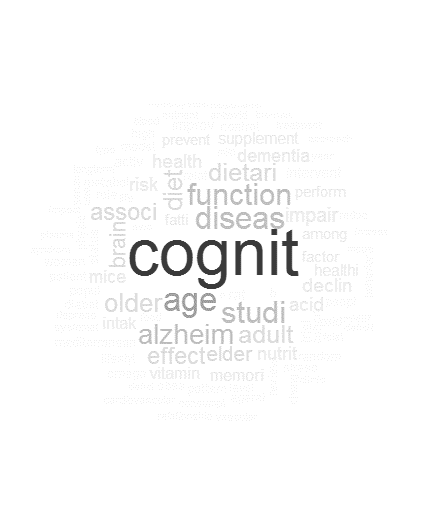 |  | dash(9.78)  scottish(8.87)  initi* (8.43) |
| **1aaa: n=3327, topic of Azheimer’s disease in ageing populations.** McKhann, G., Drachman, D., Folstein, M., Katzman, R., Price, D., & Stadlan, E. M. (1984). Clinical diagnosis of Alzheimer's disease Report of the NINCDS‐ADRDA Work Group* under the auspices of Department of Health and Human Services Task Force on Alzheimer's Disease. Neurology, 34(7), 939-939. doi: 10.1212/WNL.34.7.939 |  | 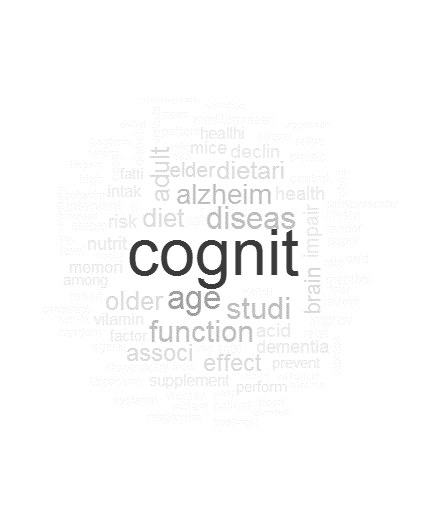 |  | depressivelik* (10.1)  alzheimerlik* (8.81)  faux(8.79) |
| **1aab: n=15, topic of dementia in developing countries.** Prince, M., Acosta, D., Chiu, H., Scazufca, M., Varghese, M., & 10/66 Dementia Research Group. (2003). Dementia diagnosis in developing countries: a cross-cultural validation study. The Lancet, 361(9361), 909-917. doi: 10.1016/S0140-6736(03)12772-9 |  | 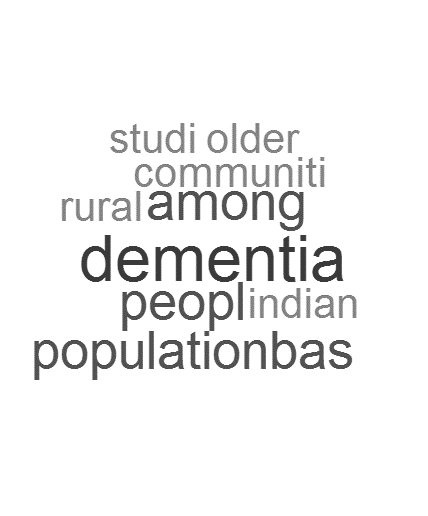 |  | franc* (10.94)  hypometabol* (9.7)  deposit* (8.95) |
| **1ab: n=80, topic of the vitamin D and cognition.** Holick, M. F. (2007). Vitamin D deficiency. New England Journal of Medicine, 357(3), 266-281. Doi: 10.1056/NEJMra070553 |  | 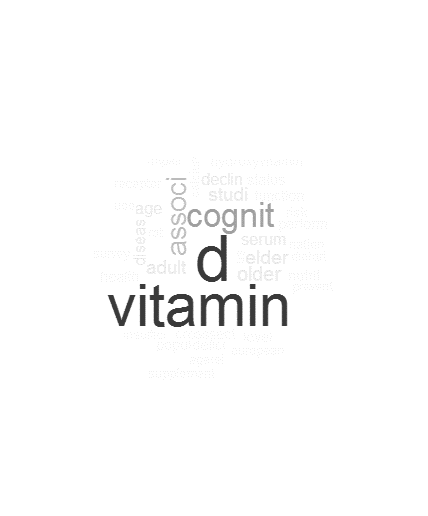 |  | fortif* (9.49)  graft (9.48)  demograph* (9.19) |
| **1b: *n*=100, topic of kidney function and cognition.** Cockcroft, D. W., & Gault, H. (1976). Prediction of creatinine clearance from serum creatinine. Nephron, 16(1), 31-41. doi: 10.1159/000180580 |  | 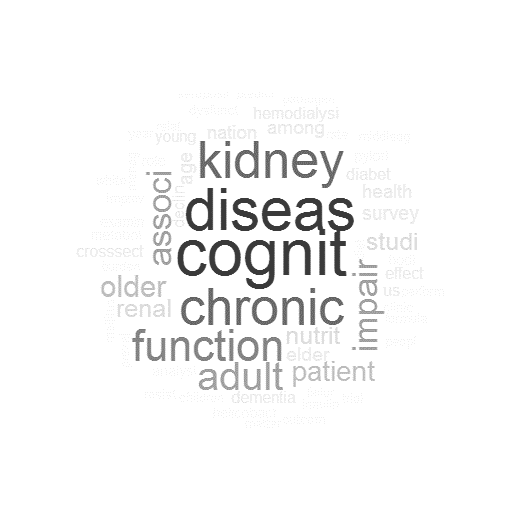 |  | wellb* (11.22) cytokine* (8.93) occlus* (7.95) westerntyp* (7.48) systemat* (7.1) |
| **1c: *n*=64, topic of antioxidants and cognition.** Milgram, N. W., Head, E., Muggenburg, B., Holowachuk, D., Murphey, H., Estrada, J., ... & Cotman, C. W. (2002). Landmark discrimination learning in the dog: effects of age, an antioxidant fortified food, and cognitive strategy. Neuroscience & Biobehavioral Reviews, 26(6), 679-695. doi: 10.1016/S0149-7634(02)00039-8 |  | 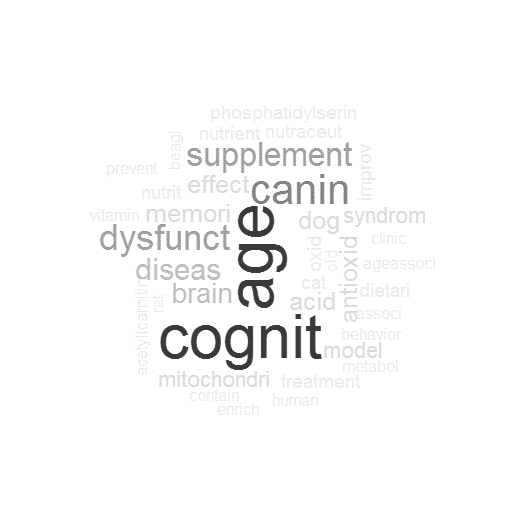 |  | highintens* (8.2) genderspecif* (7.87) trelong (7.84) hci (7.38) previous (7.36) |
| **1d: *n*=44, topic of choline and cognition.** Meck, W. H., & Williams, C. L. (2003). Metabolic imprinting of choline by its availability during gestation: implications for memory and attentional processing across the lifespan. Neuroscience & Biobehavioral Reviews, 27(4), 385-399. doi: 10.1016/S0149-7634(03)00069-1 |  | 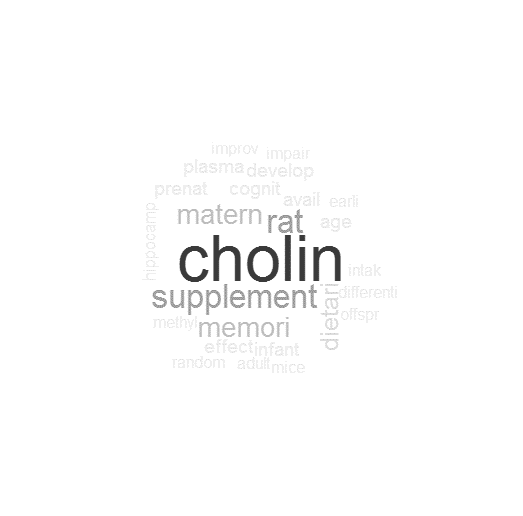 |  | fabri* (166.71) unpack (162.15) mg (162.15) odour (161.99) seroposit* (161.97) |

*Note.* A= schematic overview of citation cluster, low citation counts omitted for readability. Colored nodes indicate core citations, lines between nodes show strong citation links. Year range indicates most frequent period of publication. B= word clouds are based on raw word frequency, with larger size and opacity indicating higher frequency. β log ratio are derived from Latent Dirichlet Allocation is a Bayesian topic models. * indicates word stem wildcard. Counts in sub-clusters may exceed total cluster count, as single publications can belong to multiple clusters.

**Supplementary Table 2.** Cluster 2: *n*= 1607 publications, topic of daily self-care and nutrition in older age

| 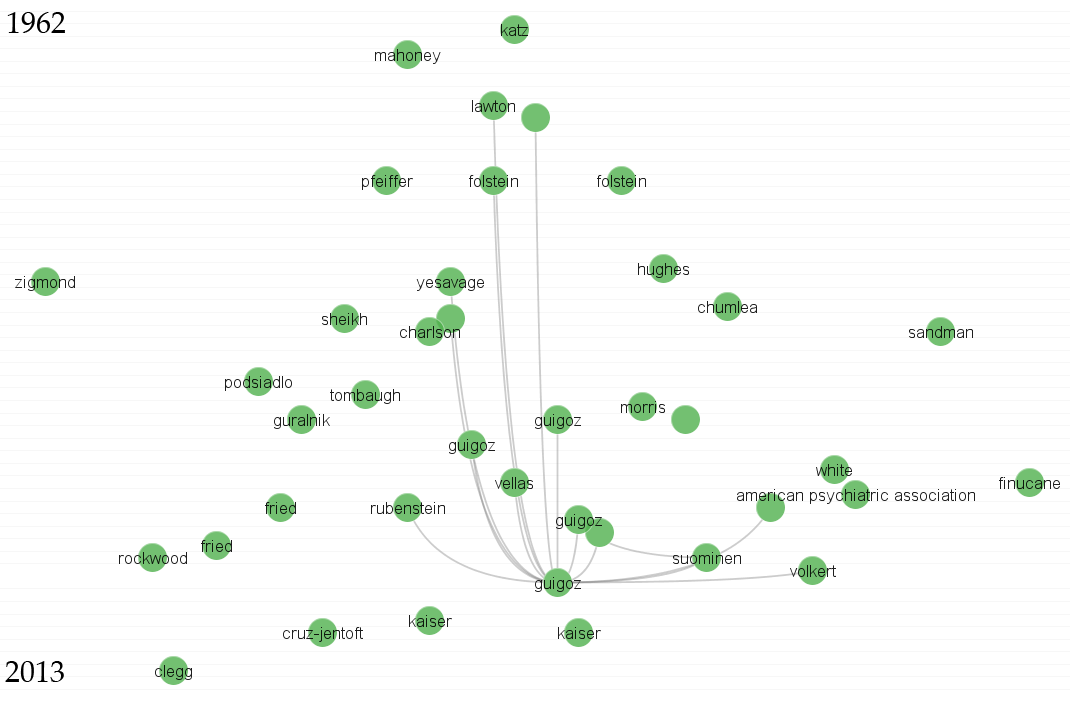 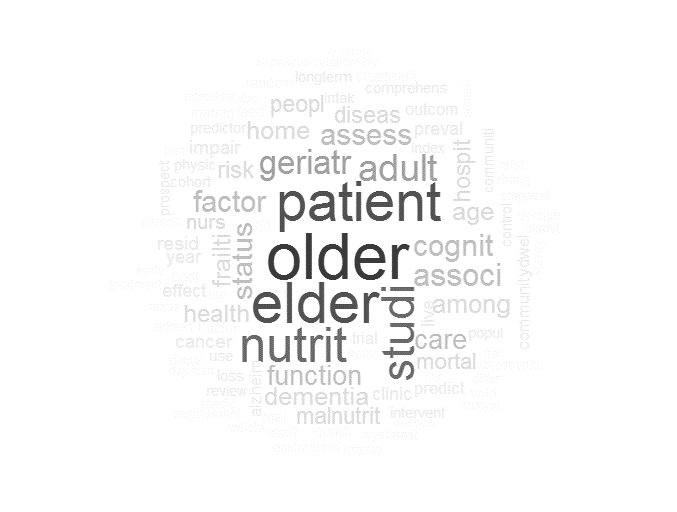 | | | | |  |
| --- | --- | --- | --- | --- | --- |
| **Cluster information and indicative core publication (weighted for recency)** |  | **Word cloud** |  | **Most unique topic terms (β log ratio)** | |
| **2a: *n*=1453, topic of daily self-care in older age** Lawton, M. P., & Brody, E. M. (1969). Assessment of older people: self-maintaining and instrumental activities of daily living. The gerontologist, 9(3_Part_1), 179-186. doi: 10.1093/geront/9.3_Part_1.179 |  | 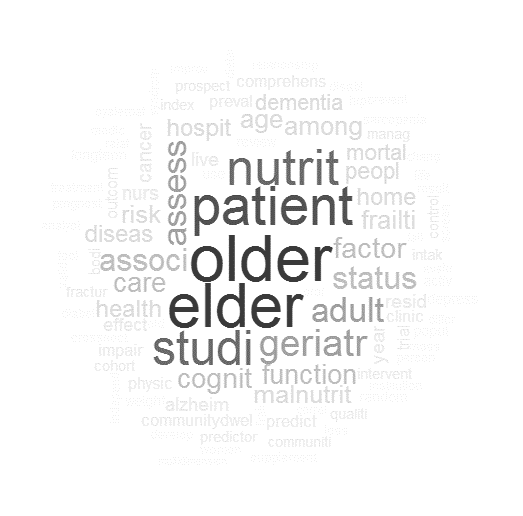 |  | dryness (352.61)  geneenviron* (352.18)  cpreval* (351.95)  english (351.76)  masticatori* (351.31) | |
| **2aa: n=1209, topic of diet and frailty in older age.** Fried, L. P., Tangen, C. M., Walston, J., Newman, A. B., Hirsch, C., Gottdiener, J., ... & McBurnie, M. A. (2001). Frailty in older adults: evidence for a phenotype. The Journals of Gerontology Series A: Biological Sciences and Medical Sciences, 56(3), M146-M157. doi: 10.1093/gerona/56.3.M146 |  | 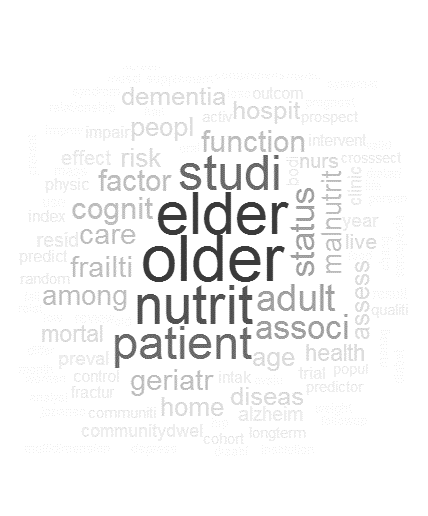 |  | standard(33.12) propens* (29.47)  sever* (29.24) | |
| **2ab: n=159, topic of cancer treatment in older age.** Repetto, L., Fratino, L., Audisio, R. A., Venturino, A., Gianni, W., Vercelli, M., ... & Aapro, M. S. (2002). Comprehensive geriatric assessment adds information to Eastern Cooperative Oncology Group performance status in elderly cancer patients: an Italian Group for Geriatric Oncology Study. Journal of clinical oncology, 20(2), 494-502. doi: 10.1200/JCO.20.2.494 |  | 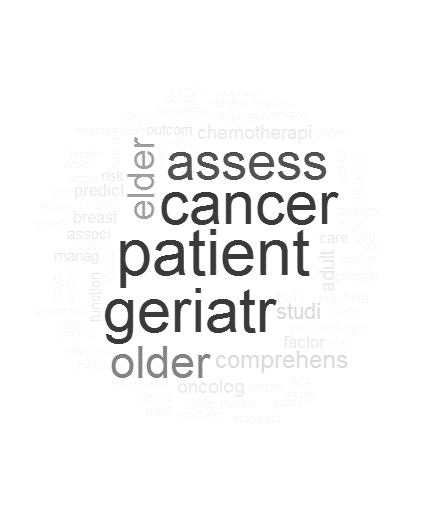 |  | technic* (297.21)  gineco* (297.12)  haematology (297.09) | |
| **2b: *n*=155, topic of feeding hospitalized elderly with dementia.** Finucane, T. E., Christmas, C., & Travis, K. (1999). Tube feeding in patients with advanced dementia: a review of the evidence. Jama, 282(14), 1365-1370. 10.1001/jama.282.14.1365 |  | 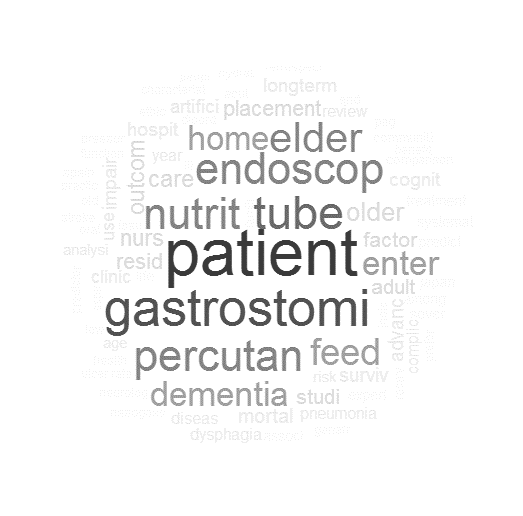 |  | glycaem* (34.93)  gerontopol* (34.81)  mindfulnessbas* (34.62)  malnutrit* (32.87)  proton (32.82) | |
| **2c: *n*=68, topic of hearing loss and dementia.** Lin, F. R., Metter, E. J., O’brien, R. J., Resnick, S. M., Zonderman, A. B., & Ferrucci, L. (2011). Hearing loss and incident dementia. Archives of neurology, 68(2), 214-220. 10.1001/archneurol.2010.362 |  | 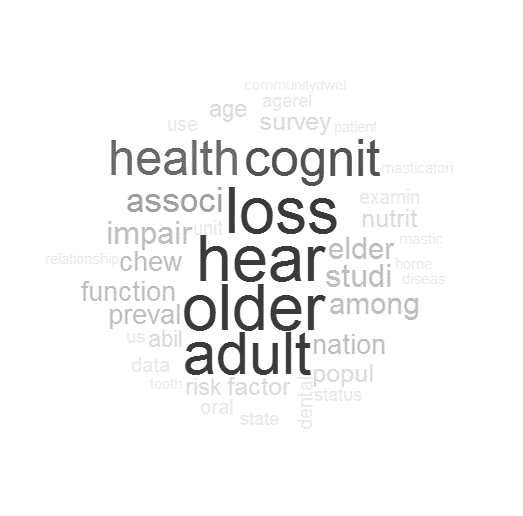 |  | confirm (52.47)  anthropometri* (50.79)  disclosur* (50.56)  suffoc* (50.5)  pathologist (50.01) | |
| **2d: *n*=10, topic of psychopathology in adults with obesity** Docet, M. F., Larranaga, A., Fernandez Sastre, J. L., & Garcia-Mayor, R. V. (2010). High rate of attention deficit hyperactivity disorder in obese adults: A case-control study. Obesity and Metabolism, 6(4), 121-124. |  | 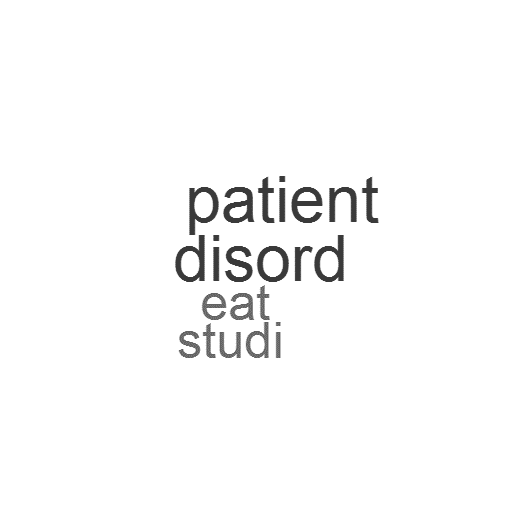 |  | glp (54.55)  proxim* (54.09)  hyperact* (53.71)  attent* (51.91)  cna* (51.84) | |

*Note.* A= schematic overview of citation cluster, low citation counts omitted for readability. Colored nodes indicate core citations, lines between nodes show strong citation links. Year range indicates most frequent period of publication. B= word clouds are based on raw word frequency, with larger size and opacity indicating higher frequency. β log ratio are derived from Latent Dirichlet Allocation is a Bayesian topic models. * indicates word stem wildcard. Counts in sub-clusters may exceed total cluster count, as single publications can belong to multiple clusters.

**Supplementary Table 3.** Cluster 3: *n*= 1542 publications, topic of the association between nutrition in early life and subsequent cognition.

| A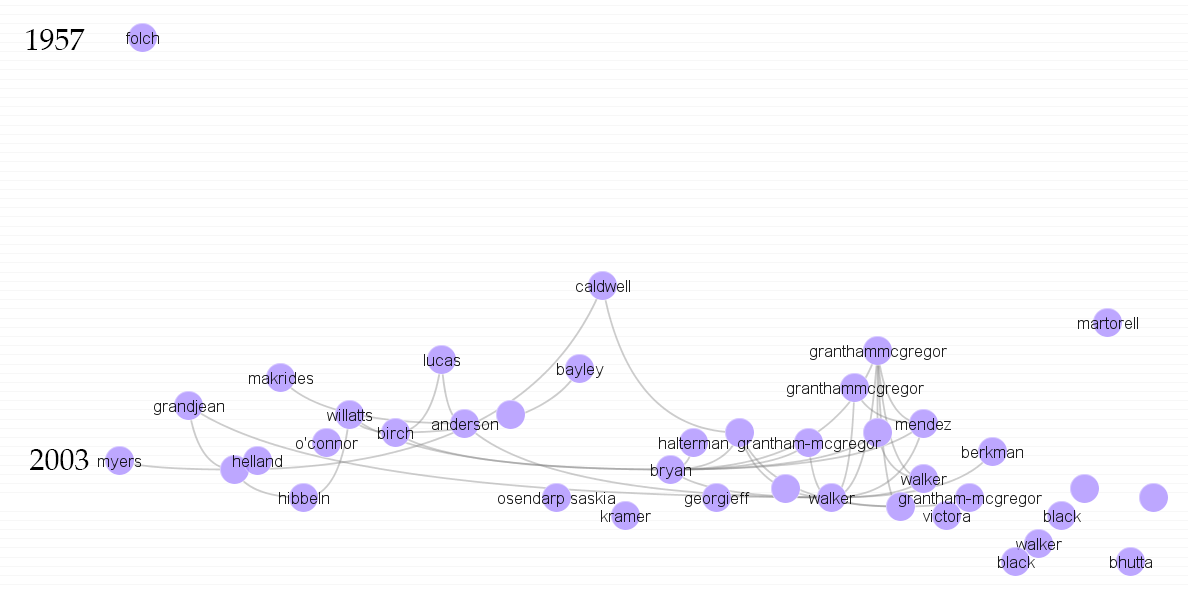 B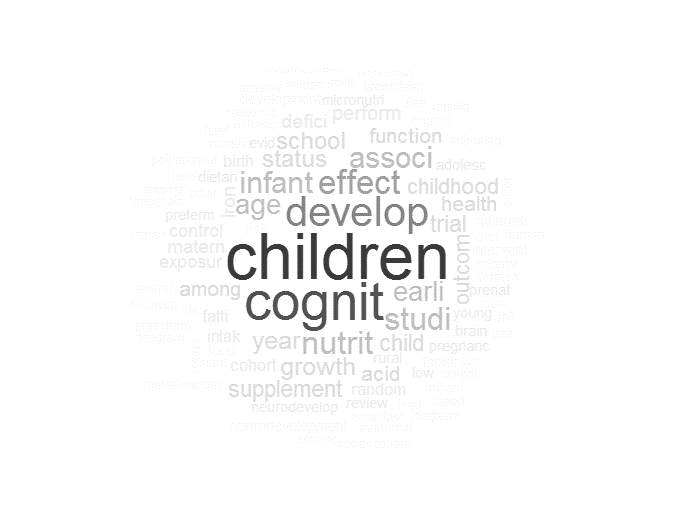 | | | | |
| --- | --- | --- | --- | --- |
| **Cluster information and indicative core publication (weighted for recency)** |  | **Word cloud** |  | **Most unique topic terms (β log ratio)** |
| **3a: *n*=927, topic of child cognitive outcomes in developing countries.** Walker, S., Wachs, T. D., Gardner, J. M., Lozoff, B., Wasserman, G. A., Pollitt, E., & Carter, J. A. Child Development in Developing Countries 2: Child development: risk factors for adverse outcomes in development countries. Lancet 369(January), 145-157. 2007. |  | 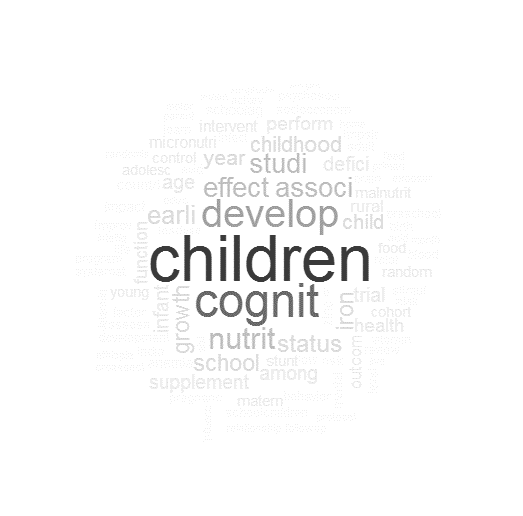 |  | data (606.25)  malnutrit* (606.22)  beyond (105.8)  anthropometr* (105.79)  adopt (105.77)  north (105.74) |
| **3aa: *n*=790, topic of child cognitive development in developing countries.** Grantham-McGregor, S., Cheung, Y. B., Cueto, S., Glewwe, P., Richter, L., Strupp, B., & International Child Development Steering Group. (2007). Developmental potential in the first 5 years for children in developing countries. The lancet, 369(9555), 60-70. doi: 10.1016/S0140-6736(07)60032-4 |  | 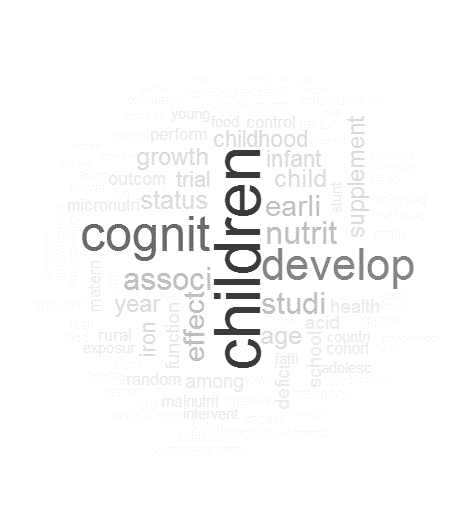 |  | shantytown (71.57)  multipl* (69.36)  sinus (69.21)  tanzanian (69.13) |
| **3aaa: *n*=770, topic of early nutrition and cognitive development.** Victora, C. G., Adair, L., Fall, C., Hallal, P. C., Martorell, R., Richter, L., ... & Maternal and Child Undernutrition Study Group. (2008). Maternal and child undernutrition: consequences for adult health and human capital. The lancet, 371(9609), 340-357. doi: 10.1016/S0140-6736(07)61692-4 |  | 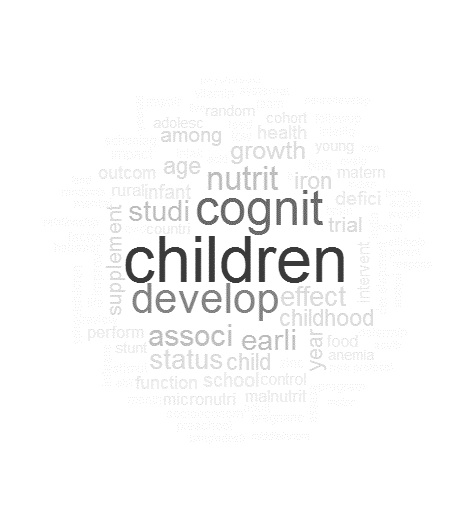 |  | activ* (9.21)  bottlefeed (7.35) development (6.07) |
| **3aab: *n*=14, topic of trace metals and cognition in children.** Menezes-Filho, J. A., Novaes, C. D. O., Moreira, J. C., Sarcinelli, P. N., & Mergler, D. (2011). Elevated manganese and cognitive performance in school-aged children and their mothers. Environmental research, 111(1), 156-163. doi: 10.1016/j.envres.2010.09.006 |  | 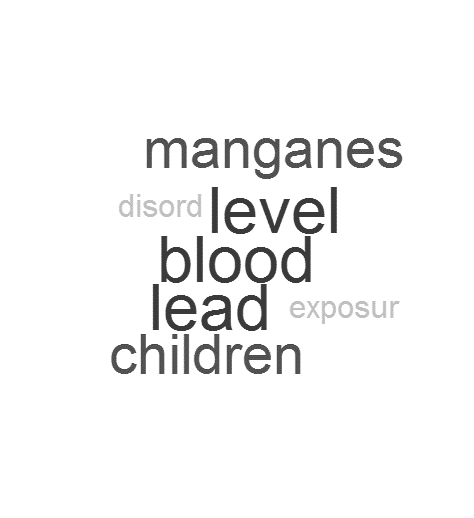 |  | sinus (9.34)  mid* (8.21)  face (7.03) |
| **3ab: *n*=72, topic of the association between breast milk and later cognition in pre-term infants.** Lucas, A., Morley, R., Cole, T. J., Lister, G., & Leeson-Payne, C. (1992). Breast milk and subsequent intelligence quotient in children born preterm. The Lancet, 339(8788), 261-264. doi: 10.1016/0140-6736(92)91329-7 |  | 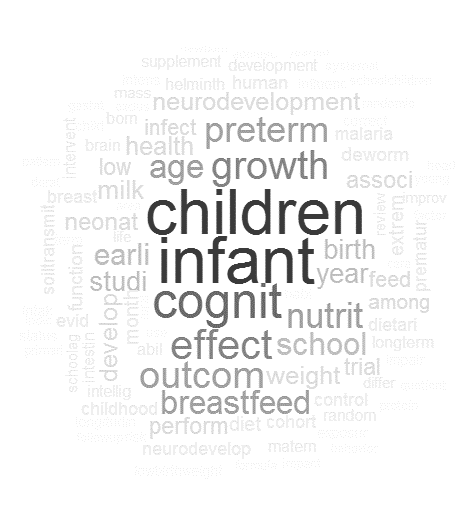 |  | formulafeed (70.02)  highdos* (87.67)  lanka (84.67)  pretermbirth (83.91)  respiratori* (83.8) |
| **3ac: *n*=25, topic of parental smoking and child cognitive outcomes.** Ogden, C. L., Kuczmarski, R. J., Flegal, K. M., Mei, Z., Guo, S., Wei, R., ... & Johnson, C. L. (2002). Centers for Disease Control and Prevention 2000 growth charts for the United States: improvements to the 1977 National Center for Health Statistics version. Pediatrics, 109(1), 45-60. doi: 10.1542/peds.109.1.45 |  | 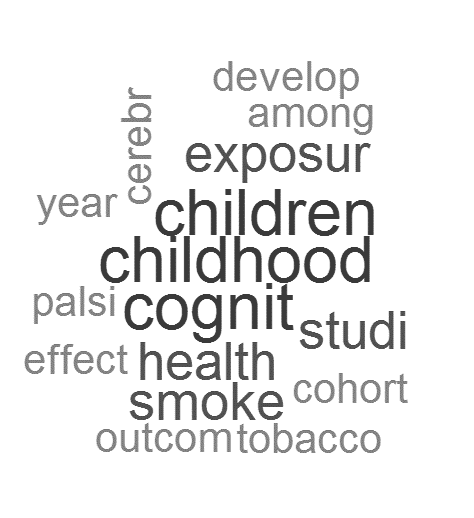 |  | reliabl* (70.8)  protest (70.41)  secular (70.18) |
| **3b: *n*=507, topic of breast feeding and cognitive outcomes.** Anderson, J. W., Johnstone, B. M., & Remley, D. T. (1999). Breast-feeding and cognitive development: a meta-analysis–. The American journal of clinical nutrition, 70(4), 525-535. doi: 10.1093/ajcn/70.4.525 |  | 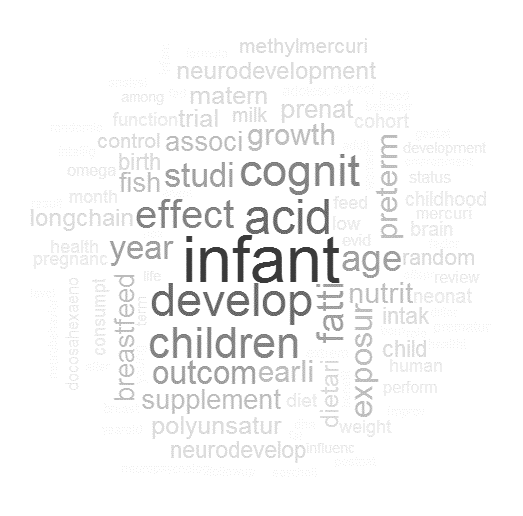 |  | stunt (72.77)  programm* (72.71)  extern* (37.97)  protect (37.32)  folat* (36.5) |
| **3ba: *n*=282, topic of maternal diet in breast feeding and infant outcomes.** Hibbeln, J. R., Davis, J. M., Steer, C., Emmett, P., Rogers, I., Williams, C., & Golding, J. (2007). Maternal seafood consumption in pregnancy and neurodevelopmental outcomes in childhood (ALSPAC study): an observational cohort study. The Lancet, 369(9561), 578-585. doi: 10.1016/s0140-6736(07)60277-3 |  | 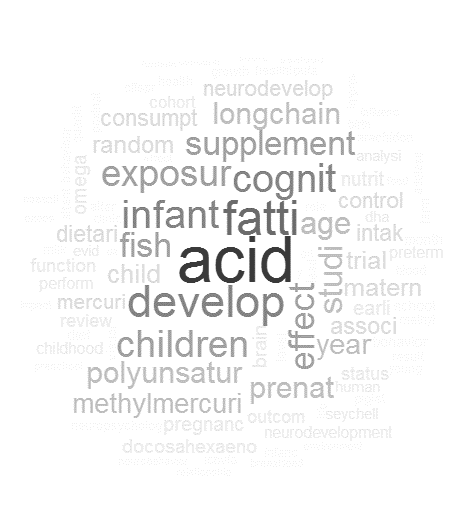 |  | dummi* (102.06)  lowincom* (101.21)  entranc* (100.02) |
| **3bb: *n*=190, topic of breast feeding and cognitive outcomes.** Kramer, M. S., Aboud, F., Mironova, E., Vanilovich, I., Platt, R. W., Matush, L., ... & Collet, J. P. (2008). Breastfeeding and child cognitive development: new evidence from a large randomized trial. Archives of general psychiatry, 65(5), 578-584. doi: 10.1001/archpsyc.65.5.578 |  | 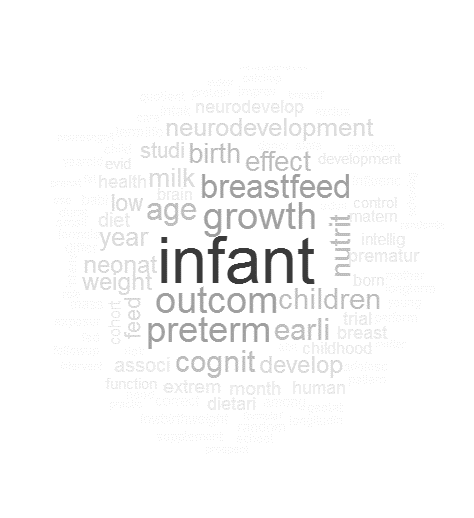 |  | folat* (92.99)  male (91.68)  formulafeed (91.65) |
| **3c: *n*=91, topic of breakfast habits in children and cognitive outcomes.** Rampersaud, G. C., Pereira, M. A., Girard, B. L., Adams, J., & Metzl, J. D. (2005). Breakfast habits, nutritional status, body weight, and academic performance in children and adolescents. Journal of the American Dietetic Association, 105(5), 743-760. doi:10.1016/j.jada.2005.02.007 |  | 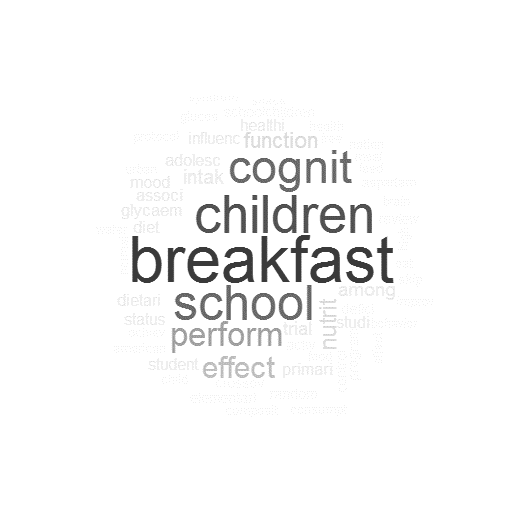 |  | monnieri (42.49)  mediterranean (42.1)  nonlinear (40.08)  limit (39.48)  oil (39.09) |
| **3d: *n*=36, topic of iodine deficiency in children and cognitive outcomes.** Bleichrodt, N., & Born, M. P. (1994). A meta-analysis of research on iodine and its relationship to cognitive development. The damaged brain of iodine deficiency, 1994, 195-200. |  | 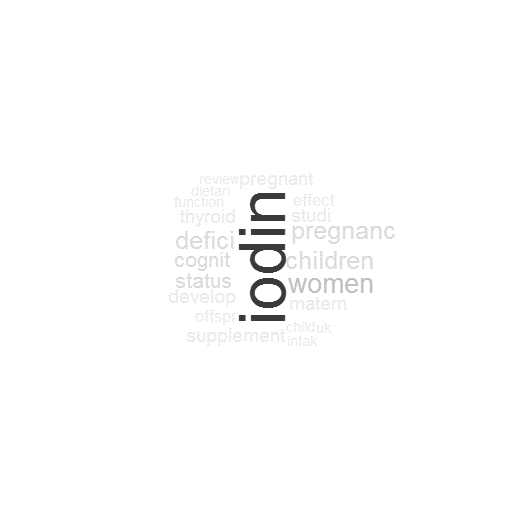 |  | iodiz* (614.52)  perchlor* (614.52)  urinari* (614.52)  iodin* (613.71)  decad* (613.52) |
| **3e: *n*=34, topic of child height as a predictor of cognitive function.** Abbott, R. D., White, L. R., Ross, G. W., Petrovitch, H., Masaki, K. H., Snowdon, D. A., & Curb, J. D. (1998). Height as a marker of childhood development and late-life cognitive function: the Honolulu–Asia Aging Study. Pediatrics, 102(3), 602-609.10.1542/peds.102.3.602 |  | 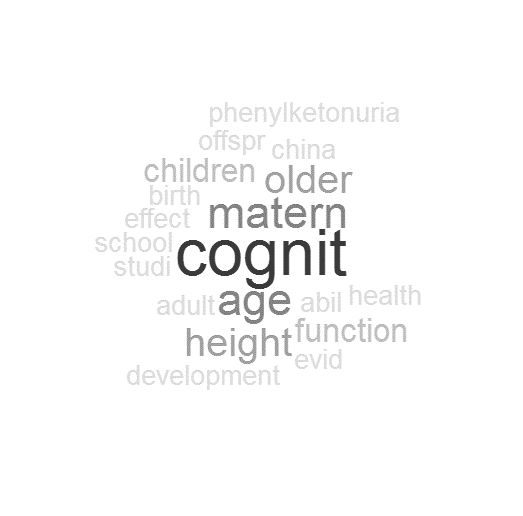 |  | anaem* (73.76)  arithmet* (73.76)  aspart* (73.76)  aspartam* (73.76)  atkin (73.76) |

*Note.* A= schematic overview of citation cluster, low citation counts omitted for readability. Colored nodes indicate core citations, lines between nodes show strong citation links. Year range indicates most frequent period of publication. B= word clouds are based on raw word frequency, with larger size and opacity indicating higher frequency. β log ratio are derived from Latent Dirichlet Allocation is a Bayesian topic models. * indicates word stem wildcard. Counts in sub-clusters may exceed total cluster count, as single publications can belong to multiple clusters.

**Supplementary Table 4.** Cluster 4: *n*= 456 publications, topic of the role of diet in overweight and obesity in children and adolescents.

| A 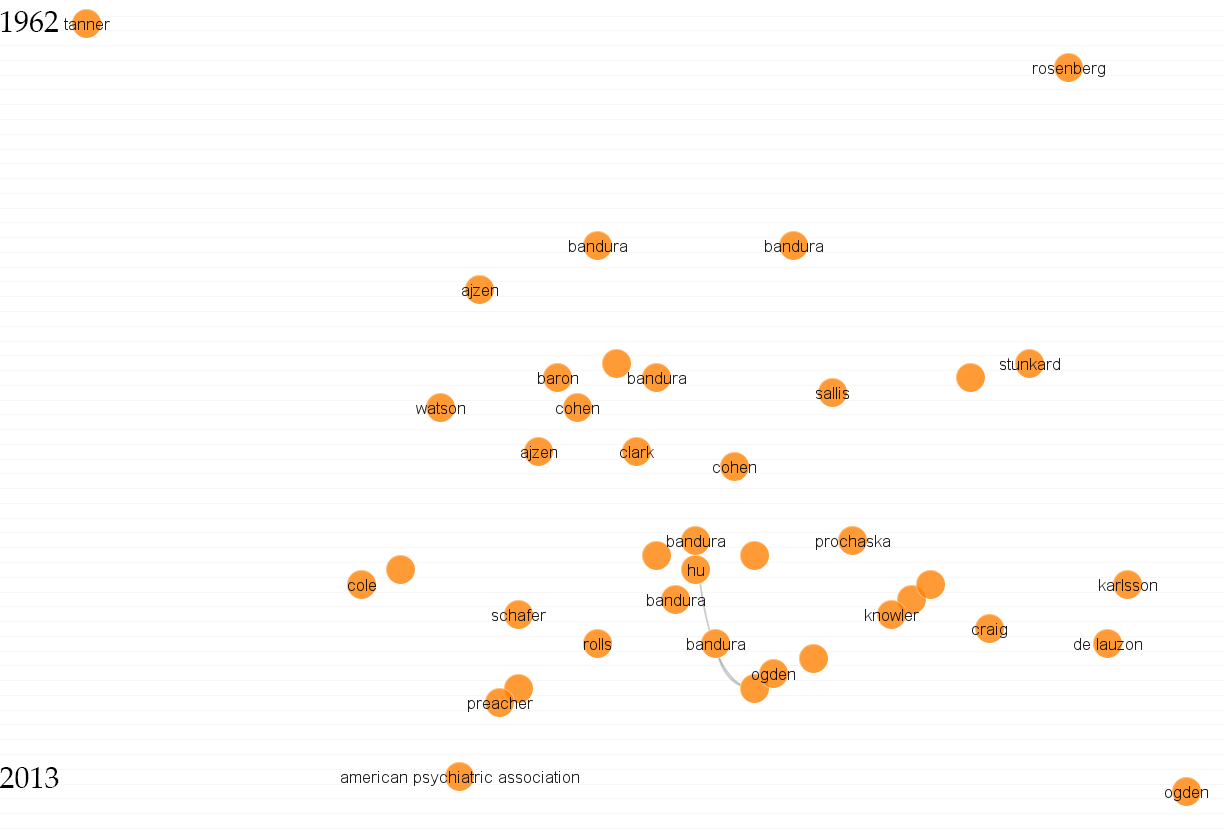 B 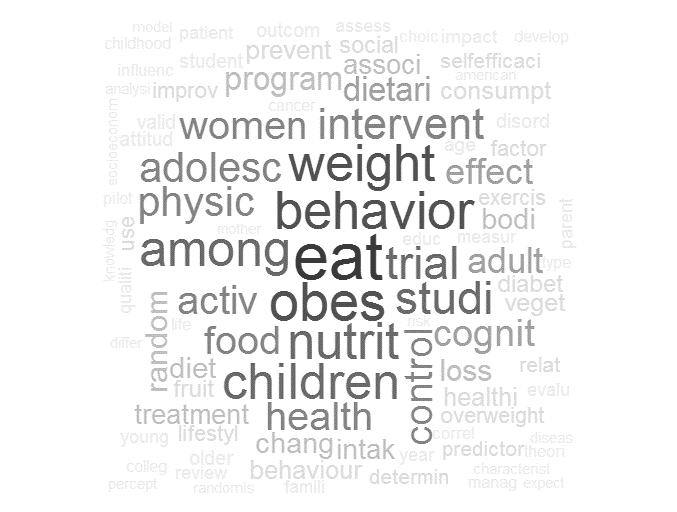 | | | | |  |
| --- | --- | --- | --- | --- | --- |
| **Cluster information and indicative core publication (weighted for recency)** |  | **Word cloud** |  | **Most unique topic terms (β log ratio)** | |
| **4a: *n*=267, topic of health promotion throughout life.** Bandura, A. (2004). Health promotion by social cognitive means. Health education & behavior, 31(2), 143-164. doi:10.1177/1090198104263660 |  | 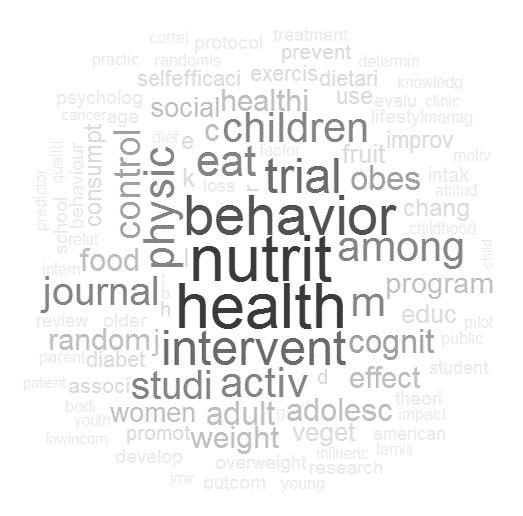 |  | al* (331.77) socialcognit* (331.73) start (330.35) media (330.02) mf* (329.92) | |
| **4b: *n*=153, topic of dietary restraint and weight loss.** Stunkard, A. J., & Messick, S. (1985). The three-factor eating questionnaire to measure dietary restraint, disinhibition and hunger. Journal of psychosomatic research, 29(1), 71-83. doi:10.1016/0022-3999(85)90010-8 |  | 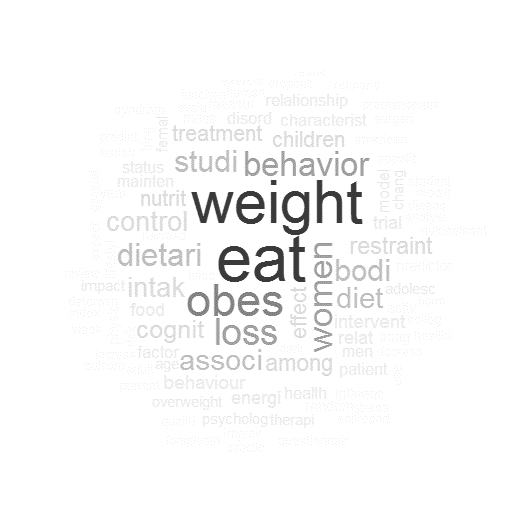 |  | evasion (617.14) fluid (617.14) cardiometabol* (616.14) chimpanzee (616.14) computertailor*(616.14) | |
| **4c: *n*=34, topic of obesity and weight loss interventions in children and adolescence.** Cole, T. J., Bellizzi, M. C., Flegal, K. M., & Dietz, W. H. (2000). Establishing a standard definition for child overweight and obesity worldwide: international survey. Bmj, 320(7244), 1240. doi:10.1136/bmj.320.7244.1240 |  | 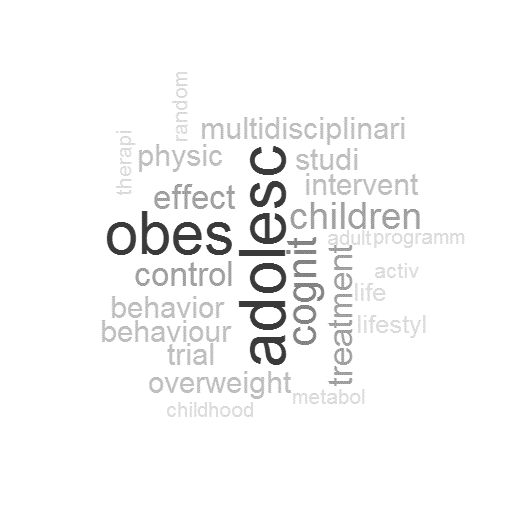 |  | energi* (611.14) characterist* (327.36) student (326.27) loss (325.92) predict (325.88) | |
| **4d: *n*=10, topic of the impact of parental nutrition on children’s health.** Wilburn, J., McKenna, S. P., Heaney, A., Rouse, M., Taylor, M., Culkin, A., ... & Lal, S. (2018). Development and validation of the Parenteral Nutrition Impact Questionnaire (PNIQ), a patient-centric outcome measure for Home Parenteral Nutrition. Clinical Nutrition, 37(3), 978-983. |  | 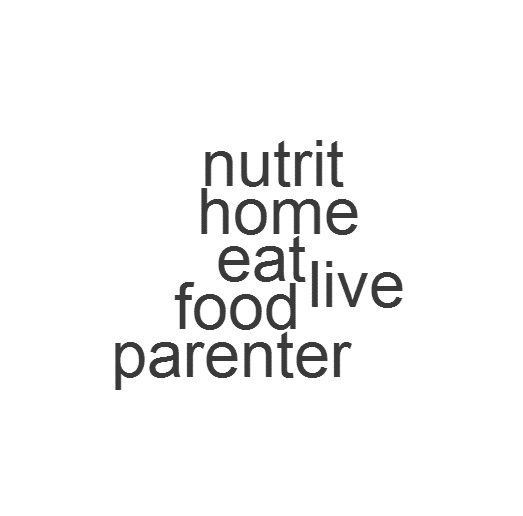 |  | parenter* (521.87) inuit* (521.29) addict* (520.29) arctic (520.29) cdetermin* (520.29) | |

*Note.* A= schematic overview of citation cluster, low citation counts omitted for readability. Colored nodes indicate core citations, lines between nodes show strong citation links. Year range indicates most frequent period of publication. B= word clouds are based on raw word frequency, with larger size and opacity indicating higher frequency. β log ratio are derived from Latent Dirichlet Allocation is a Bayesian topic models. * indicates word stem wildcard. Counts in sub-clusters may exceed total cluster count, as single publications can belong to multiple clusters.

**Supplementary Table 5.** Cluster 5: *n*= 33 publications, topic of diet and phenylketonuria

| A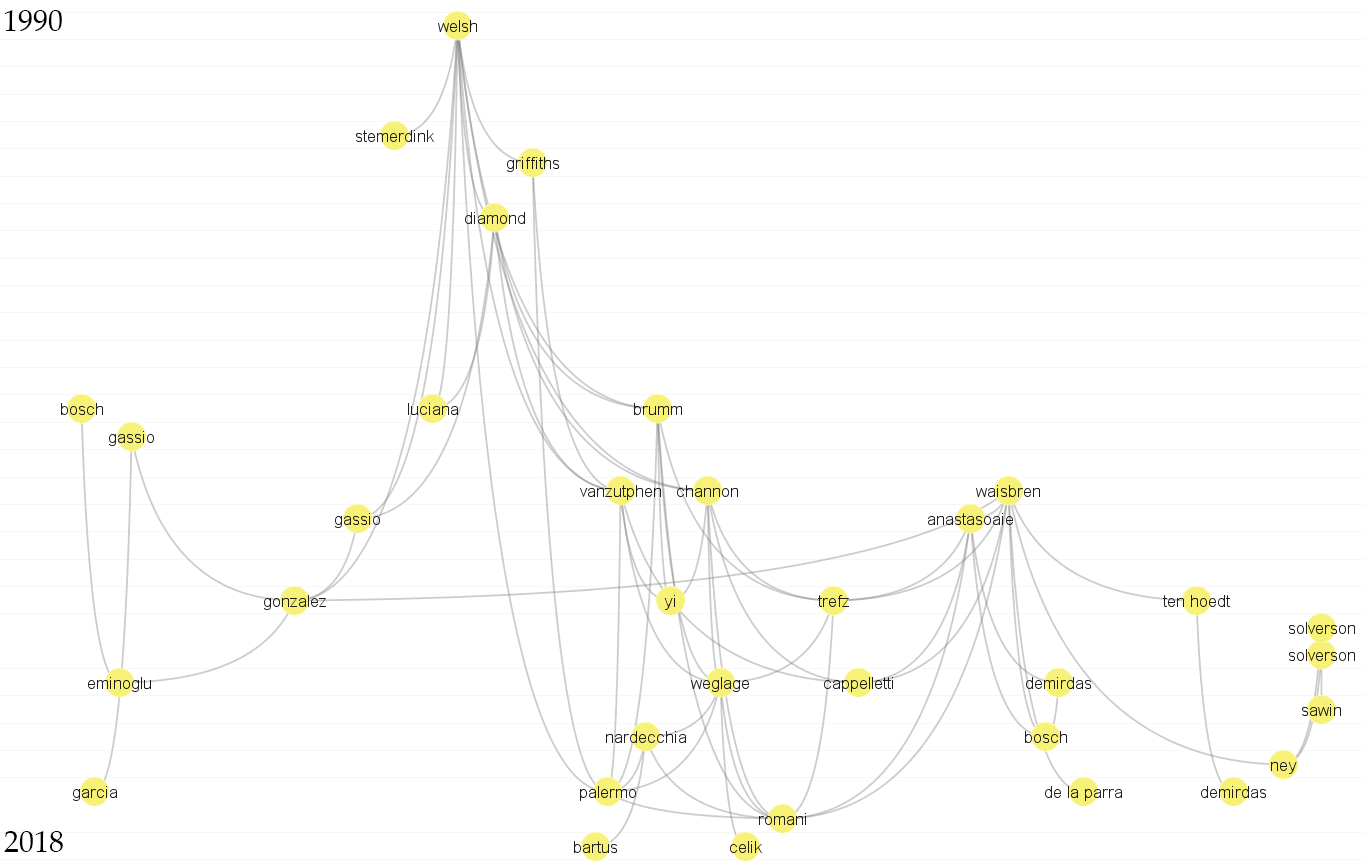 B 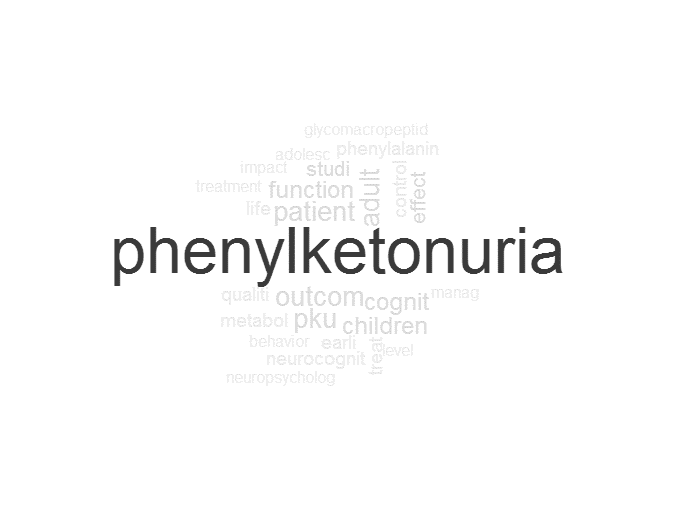 | | |
| --- | --- | --- |
| **Five indicative publications** |  | **Most unique topic terms (β log ratio)** |
| Waisbren, S. E., Noel, K., Fahrbach, K., Cella, C., Frame, D., Dorenbaum, A., & Levy, H. (2007). Phenylalanine blood levels and clinical outcomes in phenylketonuria: a systematic literature review and meta-analysis. Molecular genetics and metabolism, 92(1), 63-70. doi:10.1016/j.ymgme.2007.05.006 |  | cohort (4.212) |
| Welsh, M. C., Pennington, B. F., Ozonoff, S., Rouse, B., & McCabe, E. R. (1990). Neuropsychology of early‐treated phenylketonuria: Specific executive function deficits. Child development, 61(6), 1697-1713. doi:10.2307/1130832 |  | femal (3.703) |
| Brumm, V. L., Azen, C., Moats, R. A., Stern, A. M., Broomand, C., Nelson, M. D., & Koch, R. (2004). Neuropsychological outcome of subjects participating in the PKU adult collaborative study: a preliminary review. Journal of inherited metabolic disease, 27(5), 549-566. doi:10.1023/b:boli.0000042985.02049.ff |  | age (3.622) |
| Channon, S., Goodman, G., Zlotowitz, S., Mockler, C., & Lee, P. J. (2007). Effects of dietary management of phenylketonuria on long-term cognitive outcome. Archives of Disease in Childhood, 92(3), 213-218. doi:10.1136/adc.2006.104786 |  | long* (3.425) |
| Anastasoaie, V., Kurzius, L., Forbes, P., & Waisbren, S. (2008). Stability of blood phenylalanine levels and IQ in children with phenylketonuria. Molecular genetics and metabolism, 95(1), 17-20. doi:10.1016/j.ymgme.2008.06.014 |  | studi* (3.106) |

*Note.* A= schematic overview of citation cluster, low citation counts omitted for readability. Colored nodes indicate core citations, lines between nodes show strong citation links. Year range indicates most frequent period of publication, rather than full time period in which any publications were generated. Upper right is a word cloud based on raw word frequency, with larger size and opacity indicating higher frequency. β and β log ratio are derived from Latent Dirichlet Allocation is a Bayesian topic models. Top 5 citations reported omit repeated mention of multiple editions of the DSM. * indicates word stem wildcard.

**Supplementary table 6.** Context of the term ‘intervention’ within clusters: pairwise correlations

| **Cluster 1: the association between diet and cognitive outcomes (focus on prediction of decline and disease)** | | |
| --- | --- | --- |
| **1aaa:**  multidomain (0.46) trial (0.42) | **1aab** advanc* (r=0.99) combin* (r=0.99) dearth* (r=0.99) design* (r=0.99) elder* (r=0.99) | **1ab** independentliv* (0.99) overweight (0.99) program (0.99) cognitivebehavior (0.97) highdens* (0.97) |
| **1b** program (0.83) fast (0.79) irisin* (0.79) japanes* (0.79) kinet* (0.79) | **1c** singl* (0.77) concurr* (0.73) nutritionalbas* (0.73) proprietari* (0.73) prove (0.73) |  |
| **Cluster 2: daily self-care and nutrition in older age** | | |
| **2aa** trial (0.47) | **2ab** conven* (0.64) advoc* (0.57) attende* (0.57) conjunct (0.57) dissemin* (0.57) | **2b** adapt* (0.64) adher* (0.64) array (0.64) categor* (0.64) handl* (0.64) |
| **2c** basi* (0.64) futur* (0.53) concurr* (0.49) critic* (0.49) environ* (0.49) | **2d** ah* (0.99) ascertain (0.99) bing (0.99) blood (0.99) bmi (0.99) |  |
| **Cluster 3: the association between nutrition in early life and subsequent cognition.** | | |
| **3aaa** lay* (0.54) visitor (0.53) parentchild (0.52) advocaci* (0.51) africanamerican (0.51) | **3ab** antihelminth (0.55) divid* (0.53) biscuit (0.48) coupl* (0.48) fortif* (0.48) | **3ac** school (0.79) econom* (0.68) endogen* (0.68) equat* (0.68) link (0.68) |
| **3ba** finsteen (0.49) studiesteen (0.49) meat (0.44) mix (0.44) bergen (0.43) | **3bb** iv (0.67) babyfriend* (0.65) characteristicadjust* (0.65) cluster* (0.65) | **3c** alloc (0.66) coeduc (0.66) dine (0.66) disengag (0.66) england (0.66) |
| **3d** demograph (0.86) resourc* (0.86) anem* (0.81) assist* (0.81) capsul* (0.81) | **3e** context (0.99) normal(0.99) adapt* (0.89) environ* (0.84) absolut* (0.80) |  |
| **Cluster 4: the role of diet in overweight and obesity throughout the lifespan.** | | |
| **4b** prevent (0.66) trial (0.58) random (0.54) lifespan (0.53) deliv* (0.48) | **4c** effect (0.81) background (0.75) lifestyl* ( 0.71) glucos* (0.69) insulin (0.69) | **4d** improv* (0.99) ad* (0.94) among (0.94) arctic (0.94) avail* (0.94) |
| **Cluster 5: diet and phenylketonuria.** | percent (0.8) achenbach (0.7) adapt (0.7) allow (0.7) begun (0.7) |  |

*Note*. Figure in bracket is Pearson pairwise *r*, describing the relationship between the term “intervention” and the word reported in the table. When context within sub-clusters were identical (e.g. ‘multidomain’ had an identical 0.46 correlation for 1a, 1aa, and 1aaa), only the most specific cluster is reported. Where no pairwise correlation >0.5 was present, that cluster is omitted. * indicates word stem truncation. Correlations obtained via the findAssocs’ function of tm version 0.6-2.

**Supplementary table 7.** Context of the term ‘intervention’ within clusters: Latent Semantic Neighbourhood Analysis.

| **Cluster 1: the association between diet and cognitive outcomes (focus on prediction of decline and disease)** | | |
| --- | --- | --- |
| **1aaa:**  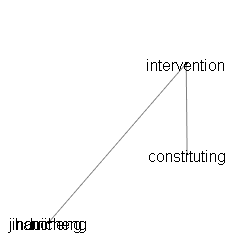 constituting (0.37) hancheng (0.35) jinduicheng (0.35) | **1ab:**  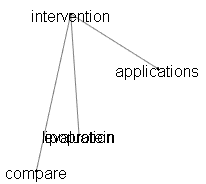applications (0.99) compare (0.99) evaluation (0.99) lipoprotein (0.99) | **1b:**  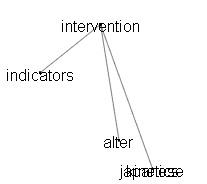 alter (0.95) indicators (0.95) japanese (0.95) kinetics (0.95) |
| **1c:**  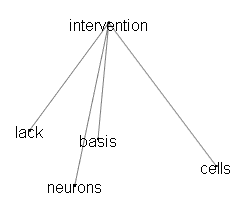  basis (0.89) lack (0.89) cells (0.87) neurons (0.84) |  |  |
| **Cluster 2: daily self-care and nutrition in older age** | | |
| **2aa:** 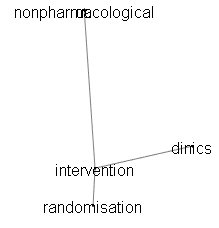randomisation (0.58) nonpharmacological (0.42) uc (0.42) dinics* (0.4) | **2ab:**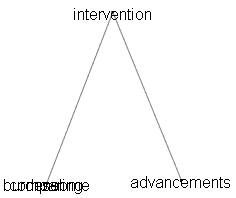 advancements (0.75) burdensome (0.75) comparing (0.75) counseling (0.75) | 2b: 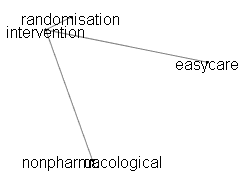  assessed (0.89) controlled (0.87) cochrane (0.87) diagnostic (0.87) |
|  |  |  |
| **2c:**  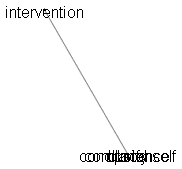 blood (0.79) clarify (0.79) competence (0.79) self (0.79) | 2d:  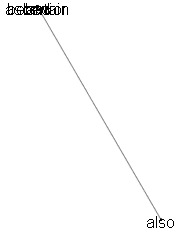 also (1) am (1) ascertain (1) bed (1) behavior (1) |  |
| **Cluster 3: the association between nutrition in early life and subsequent cognition.** | | |
| **3aaa:** 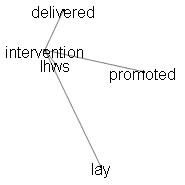 delivered (0.48) lhws (0.41) lay (0.37) promoted (0.37) | **3ab:** 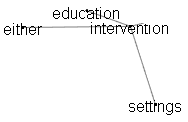  either (0.79) education (0.78) settings (0.75) | **3ac:** 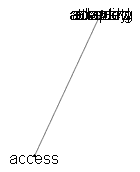  access (1) adapted (1) adversity (1) already (1) attending (1) |
| **3a:**  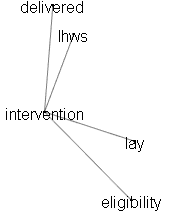 delivered (0.46) lhws (0.41) lay (0.37) eligibility (0.37) | 3ba: 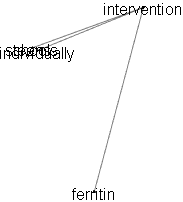  individually (0.82) ferritin (0.81) schools (0.8) teens (0.8) | **3bb:** 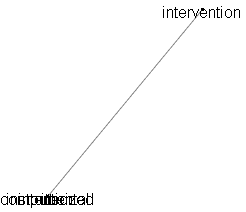 computerized (0.83) instrumental (0.83) intention (0.83) itt (0.83) |
| **3b:** 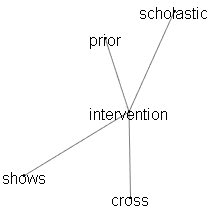 prior (0.76) cross (0.73) shows (0.7) scholastic (0.68) | **3d:** 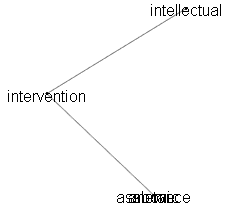 intellectual (0.96) above (0.94) anemic (0.94) assistance (0.94) | **3e:** 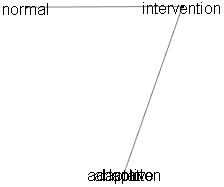  normal (1) absolute (0.91) adaptation (0.91) adaptive (0.91) |
| **Cluster 4: the role of diet in overweight and obesity in childhood and adolescence.** | | |
| **4a:** 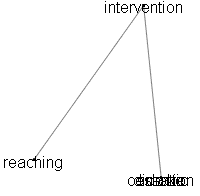 reaching (0.52) cessation (0.49) smoke (0.49) didactic (0.49) | **4b:** 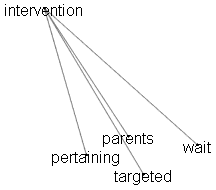 parents (0.82) wait (0.81) pertaining (0.76) targeted (0.75) | **4c:** 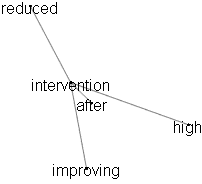 after (0.98) high (0.84) improving (0.8) reduced (0.78) |
| 4: 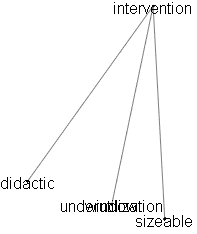 didactic (0.46) sizeable (0.46) underutilization (0.46) window (0.46) | **Cluster 5: diet and phenylketonuria.** | **5.** 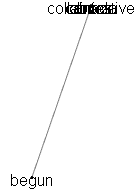 begun (1) canada (1) clinics (1) collaborative (1) context (1) |

*Note*. Figure in bracket is distance in semantic space (as pictured in the figures) between the term “intervention” and the word reported in the table. This is obtained by neighborhood analysis, a method of latent semantic analysis which takes into account the larger semantic context of the term, as derived from the text (here, corpora formed by abstracts within each cluster). Neighbourhood analysis was carried using the ‘neighbors’ function of the LSAfun package (version 0.5.1). When context within sub-clusters were identical, only the most specific cluster is reported. When there was insufficient proximity between “intervention” and other terms in the cluster for neighbourhood analysis to converge, that cluster is omitted.
